# Molecular targets and mechanisms of traditional Chinese medicine combined with chemotherapy for gastric cancer: a meta-analysis and multi-omics approach

**DOI:** 10.1080/07853890.2025.2494671

**Published:** 2025-05-03

**Authors:** Jie Lin, Jincheng Wang, Kai Zhao, Yongzhi Li, Xuewen Zhang, Jiyao Sheng

**Affiliations:** ^a^ Department of General Surgery, The Second Hospital of Jilin University, Changchun, China; ^b^ Department of Gastrointestinal Surgery, The Second Hospital of Jilin University, Changchun, China

**Keywords:** Traditional Chinese medicine (TCM), gastric cancer (GC), prognosis and biomarker, precise drug management

## Abstract

**Background:**

The combination of traditional Chinese medicine (TCM) with chemotherapy has been widely applied in the treatment of gastric cancer (GC). However, previous clinical studies have been constrained by small sample sizes and a lack of investigation into the molecular mechanisms of TCM. This study aims to assess the efficacy of TCM in treating GC by leveraging the strengths of meta-analysis and multi-omics approaches while also summarizing the underlying pharmacological mechanisms.

**Methods:**

A systematic literature review and meta-analysis were conducted using online databases to collect data before May 2024. This was to investigate the association between TCM combined with chemotherapy and the prognosis in GC. The molecular targets between the high-frequency TCMs and GC were identified through network pharmacology. The underlying mechanisms were investigated using multi-omics

**Results:**

9 studies with 2,158 patients were included. The meta-analysis results demonstrated that the combination of TCM and chemotherapy significantly improved the overall survival (OS) of GC patients (OR = 2.91; 95% CI: 2.70–3.12, *p* < 0.00001) and enhanced their quality of life (OR = 4.00; 95% CI: 1.99–8.03, *p* < 0.0001). Network pharmacology analysis identified 13 potential molecular targets of TCM in GC; additionally, multi-omics analysis highlighted the significant roles of MK, MIF, GALECTIN, and CypA signaling pathways in GC.

**Conclusion:**

The combination of TCM with chemotherapy significantly improves the prognosis of GC; future research can focus on these key molecular targets and signaling pathways. This supports the application of precision medicine in cancer treatment and suggests the rational use of TCM in managing GC.

## Introduction

Gastric cancer (GC), the fifth most common malignancy globally, is the fourth leading cause of cancer-related deaths [1]. In recent years, the incidence of GC has continued to rise, with approximately 1 million new cases diagnosed worldwide each year [2,3]. Chemotherapy has been widely used in the treatment of GC; however, studies have shown that its overall efficacy remains poor [4]. Investigations into chemotherapy outcomes for GC revealed that, even with optimal supportive care, chemotherapy extended survival by only 6–7 months; additionally, multidrug combination chemotherapy extended survival by merely 1 month compared to monotherapy [5]. Previous research conducted by our team has confirmed the therapeutic value of combining TCM with chemotherapy in liver cancer [6]. Therefore, it is imperative to further investigate the role of TCM in the chemotherapy regimen for GC. This approach has the potential to not only enhance overall patient outcomes but also to refine treatment protocols.

TCM is extensively applied in managing GC; according to TCM’s dialectical theory, the causes of GC are primarily attributed to qi stagnation and blood stasis (blocked energy flow and poor blood circulation), phlegm-dampness blockage (accumulation of mucus and fluid), heat toxicity (excessive inflammation or heat in the body), and weakness of healthy qi (weakened vitality or immune function). TCM aims to treat GC by balancing yin and yang (restoring internal harmony), tonifying deficiencies (strengthening weaknesses), draining excesses (reducing excess), regulating the zang-fu organs (improving organ function), and dredging channels and collaterals (unblocking energy pathways). Chao et al. demonstrated that the TCM treatment group had a prolonged survival rate for early GC compared to the non-TCM treatment group [7]. Similarly, Liu et al. confirmed the survival benefits of combining TCM with chemotherapy for advanced GC [8]. The limited efficacy of chemotherapy is often due to the specificity of its drug targets, which typically affect a single target or a few pathways, failing to induce systemic modulation. In contrast, the multi-target properties of TCM offer a potential solution to this challenge. Studies have demonstrated that the pharmacogenomics of TCM can modulate the function of enzymes and drug-metabolizing transporters [9], thereby providing a theoretical basis for its use as a therapeutic strategy for GC. However, some studies have raised different perspectives, noting that the compound nature of TCM decoctions might result in varying effects when combined with chemotherapy [10]. Research has shown that the application of multi-omics data can identify biomarkers for diseases like GC and inform the development of potential therapeutic strategies [11–13]. Therefore, it is essential to identify the effective components and underlying mechanisms of TCM when used in conjunction with chemotherapy for GC, underscoring the need for additional research to explore these dynamics.

Traditional experimental approaches had often failed to identify the active components and molecular targets of multiple Chinese herbal medicines. However, the burgeoning field of big biological data and advancements in bioinformatics recently kindled hope for elucidating the intricate mechanisms by which TCM functioned in the treatment of GC [14]. Exploring the potential of TCM in treating GC using multi-omics approaches is of significant importance [15,16]. This study leveraged the synergistic benefits of meta-analysis, network pharmacology, and multi-omics approaches to investigate the potential pharmacological mechanisms underlying the combination of TCM with chemotherapy.

## Materials and methods

### Data sources and search strategy

**Figure 1.**
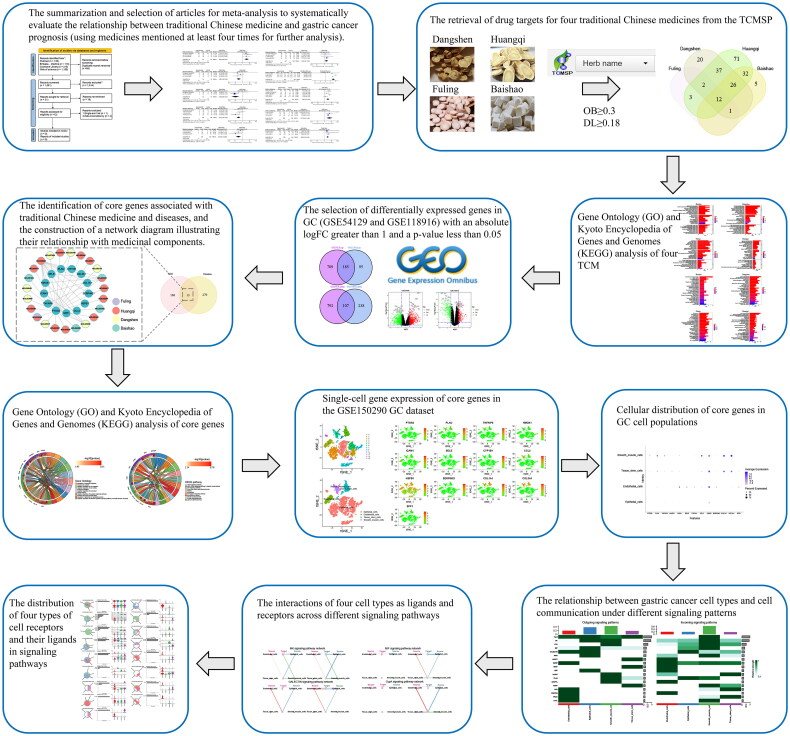
The flowchart of this study.

By May 1, 2024, two researchers, JL and JCW, conducted independent searches across various databases such as PubMed, EMBASE, Cochrane Library, and Web of Science, focusing on randomized controlled trials. They utilized medical subject headings to locate terms like ‘traditional Chinese medicine’ and ‘gastric cancer,’ including related synonyms. Furthermore, they also reviewed the full-text reference lists of pertinent studies. The research adhered to the PRISMA [17] guidelines and was documented in the PROSPERO database under the registration number CRD42024536268. The flowchart of the study, as shown in Figure 1.

### Inclusion and exclusion criteria

Two researchers independently assessed all records using predetermined inclusion and exclusion criteria and managed these records with EndNote software. After duplicates were removed, they conducted detailed reviews of full texts to identify suitable clinical trials. Inclusion criteria required patients to be diagnosed with gastric cancer *via* histological or cytological methods, have a life expectancy of over three months, and be part of a study where the intervention group received traditional Chinese medicine in addition to chemotherapy. The control group received no traditional Chinese medicine, only a placebo or standardized chemotherapy. Each study needed at least 10 patients per group and had to report at least one validated clinical outcome, such as objective response rate (ORR), disease control rate (DCR), adverse reactions, or survival rates spanning 1–5 years. Studies failing to meet these standards were excluded. Patient characteristics and major clinical outcomes analyzed in the meta-analysis are detailed in Tables 1, S1, S2, and S3.

**Table 1.** Main characteristics of included studies.

Author year	No. of patient(n)		Age (years)		Intervention measurement	
	Combination therapy	Chemotherapy	Combination therapy	Chemotherapy	Combination therapy	Chemotherapy
Hongzhen Wang 2007	34	32	55.44 ± 4.53	54.2 ± 5.35	Chemotherapy + Fuzheng Hewei Decoction	DCF
Jiemin Zhao 2007	30	30	56	55	Chemotherapy + Shenqi Fuzheng injection	Docetaxel + fluorouracil + calcium folinate
LIU Yun-xia 2009	28	19	–	–	Chemotherapy + Yiqi Bushen Koufuye	FOLFOX
Xuan Liu 2017	88	94	61.16 ± 12.43	59.57 ± 13.37	Chemotherapy + Kanglaite injection/Cinobufacini injection/Yadanzi injection/Elemene injection/Xiaoaiping injection/Xianrengu oral liquid/Jinlongshe oral liquid	DCF/ECF/XELOX/FOLFOX/FOLFIRI
Yun Luo 2017	170	132	60.58 ± 10.89	56.64 ± 11.52	Chemotherapy + Kanglaite injection/Bufasini injection/Brucea brucea injection/olive injection/Xiaoaiping injection/Xianrengu oral liquid/Jinlongsnake oral liquid	Chemotherapy
Peng Shu 2019	251	238	59 (22-75)	58 (20-75)	Chemotherapy + Yiqi huayu Jiedu decoction	DCF/FOLFOX
Li De-Hui 2020	29	28	57.36 ± 8.87	58.25 ± 9.64	Chemotherapy + Shunqi Yiwei Decoction	SOX
Chao Hou 2021	122	122	–	–	Chemotherapy + Fuzheng detoxification therapy	Chemotherapy
Qunying Zou 2021	69	76	–	–	Chemotherapy + Fuzheng Jiedu decoction	Capecitabine

DCF: Docetaxel + 5-fluorouracil + cisplatin; FOLFOX: Calcium folinate + fluorouracil + oxaliplatin; ECF: Epirubicin + cisplatin + fluorouracil; XELOX: Capecitabine + oxaliplatin; FOLFIRI: Calcium folinate + fluorouracil + irinotecan; SOX: Ticeo + oxaliplatin.

**Table 2.** The high-frequency Chinese herbs in each study.

Chinese name	Pharmaceutical name	Counts	Frequency 1 (counts/total herb counts)	Frequency 2 (counts/study numbers)
Fuling	Poria	7	3.91%	77.78%
Huangqi	Astragalus	7	3.91%	77.78%
Dangshen	Codonopsis	6	3.35%	66.67%
Baishao	Radix paeoniae alba	4	2.23%	44.44%

### Data extraction and quality assessment

Two researchers independently extracted data from the selected studies into standardized Excel spreadsheets, documenting details including the first author’s name, publication year, type of study, number of patients, ages, specifics of the intervention, and perioperative outcomes. A third researcher then confirmed the accuracy of this data; any discrepancies identified were resolved through discussion between the initial researchers. The quality of the studies was evaluated using the well-established Newcastle Ottawa Scale (NOS) (https://www.ohri.ca//programs/clinical_epidemiology/oxford.asp). This comprehensive scale assesses three key domains: the selection of study groups, the comparability between groups, and the rigor of outcome measurements. The maximum achievable score on the scale is 9 points. Based on the scores, studies were classified into three categories: low quality (3–4 points), medium quality (5–6 points), and high quality (7–9 points).

### Data collection

In this study, the GC data were sourced from the Gene Expression Omnibus (GEO) database (https://www.ncbi.nlm.nih.gov/geo/). RNA transcriptome sequencing data came from GSE54129 and GSE118916, and single-cell sequencing data came from GSE150290. Furthermore, information regarding Traditional Chinese Medicine (TCM) components and their pharmacological genes was extracted from the Traditional Chinese Medicine Systems Pharmacology Database and Analysis Platform (TCMSP, https://old.tcmsp-e.com/tcmsp.php).

### Identification of core genes in TCM-GC

The selection of TCM prescriptions for network pharmacology research was guided by their frequency of use, as determined through meta-analytical studies. Only those prescriptions applied more than three times were considered for further analysis.

Oral bioavailability (OB) measures the rate and concentration of a drug reaching systemic circulation, whereas drug-likeness (DL) assesses its similarity to established compounds. For this study, active components in TCM were identified using a minimum threshold of 30% for OB and 0.18 for DL. Following this, annotations for human gene targets of these components were performed using information obtained from The Universal Protein Database (UniProt, https://legacy.uniprot.org/).

Differential genes for GC were obtained from GSE54129 and GSE118916 using the limma package, with screening criteria set at |logFC| > 1 and *P-*value < 0.05. The VennDiagram package was employed to identify intersecting genes between TCM and GC. These data were then uploaded to Cytoscape 3.8.2 to generate a TCM-active component-target network map.

### Enrichment analysis of GO and KEGG

Functional enrichment analysis was conducted on crucial drug molecules and TCM-GC, involving Gene Ontology (GO) and Kyoto Encyclopedia of Genes and Genomes (KEGG) pathways. This analysis employed multiple packages such as clusterProfiler, org.Hs.eg.db, enrichplot, circlize, RColorBrewer, and ComplexHeatmap. The criteria for screening included a *P-*value and *Q-*value both set below 0.05.

### Cell type identification and gene localization

Recently, single-cell sequencing has become extensively employed to examine phenomena at the cellular level, facilitating rapid differentiation of cell types and localization of genes. The GSE150290 dataset underwent processing and quality control, with the data segmented into distinct cell clusters through batch effect elimination, dimensionality reduction, and cell annotation. The distribution of TCM-GC genes across these clusters was then analyzed using the Seurat and Harmony packages.

### Analysis of intercellular communication

Interactions between cells were investigated with the CellChat R package (version 1.5.0) to uncover potential communication mechanisms at the single-cell level. Intercellular communication networks were computed, and the signals from each cell cluster were characterized using the aggregateNet function in CellChat. Additionally, the computeNetSimilarity function was employed to identify signal clusters exhibiting functional or structural similarities.

### Statistical analysis

Data analysis and meta-processing utilized RevMan 5.4 software. For dichotomous variables, odds ratios (OR) served as effect size indicators, and for continuous variables, mean differences (MD) were applied, both presented with 95% confidence intervals (CI) in forest plots. When no significant heterogeneity was detected (I^2^ < 50%, *p* > 0.1), a fixed-effect model was employed for the meta-analysis. Conversely, in the presence of significant heterogeneity (I^2^ > = 50%, *P* < = 0.1), a random-effects model was utilized. To enhance the robustness of the analysis, we conducted a power analysis using PASS (version 23.0.2) on the results of the primary meta-analysis to assess whether the sample size was sufficient to detect meaningful effects (a value greater than 0.6 denotes statistical significance). Analyses of subgroups were conducted focusing on overall survival (OS) and types of adverse events. Funnel plots within the RevMan 5.4 software were used to visually evaluate publication bias.

## Results

### The characteristics of the included studies

**Figure 2.**
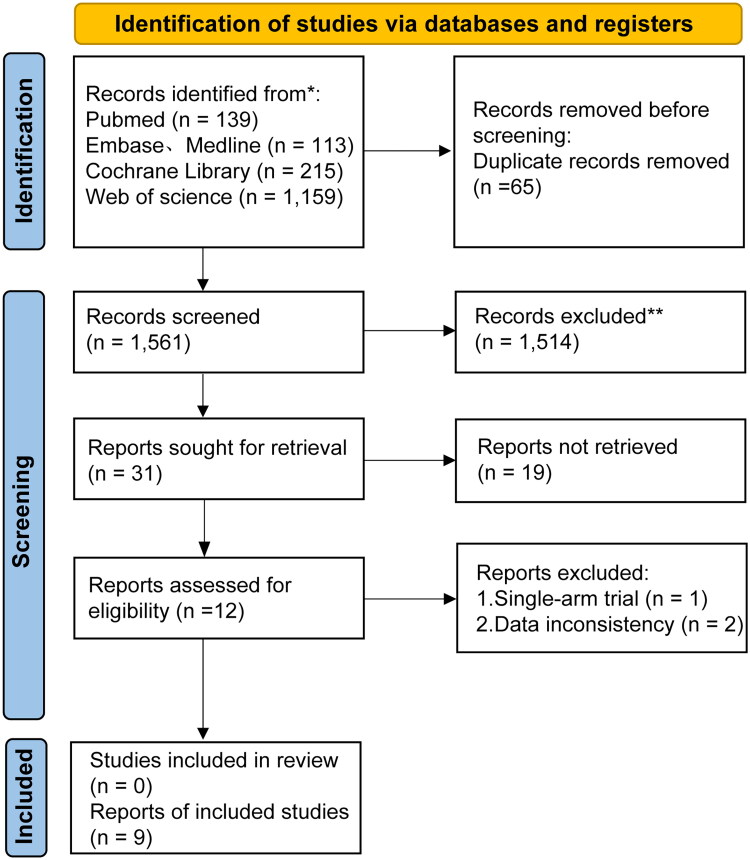
Preferred reporting items for systematic reviews and meta-analyses (PRISMA) diagram of the study selection.

Figure 2 presented the PRISMA diagram that depicted the process of study selection. From the search strategy, 1,626 publications were initially retrieved (139 from PubMed, 113 from Embase, 215 from the Cochrane Library, and 1,159 from Web of Science). Following the removal of 65 duplicate entries, 9 [8,18–25] studies involving a total of 1,592 patients were ultimately selected for inclusion in the meta-analysis. The evaluation of research quality, as assessed by the NOS, revealed that all included studies scored ≥6, indicating a moderate to high overall quality (Table S4). A visual representation of the research quality for the included studies is provided in Supplementary Figure 1.

### Tumor response and multi-survival analysis

**Figure 3.**
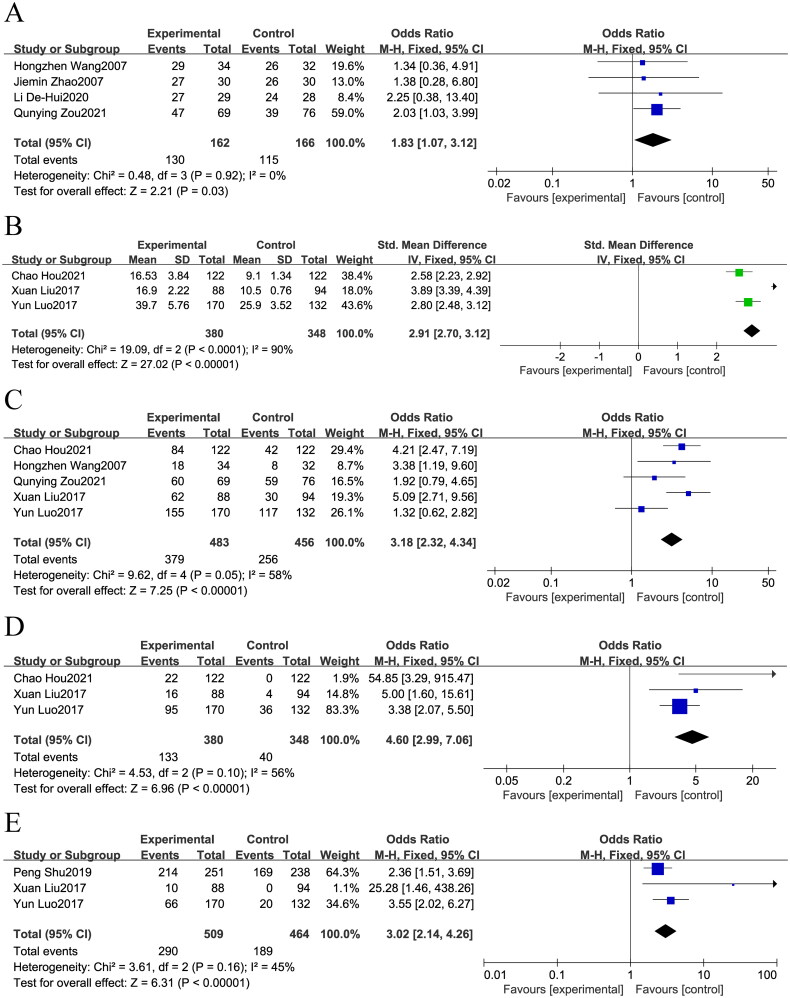
Forest plot of Chinese medicine combined with chemotherapy. (A): DCR; (B): median survival; (C): 1-year survival rate; (D): 3-year survival rate; (E): 5-year survival rate.

This study assessed the effectiveness of integrating TCM with chemotherapy in treating GC, using complete response (CR), partial response (PR), stable disease (SD), ORR, and DCR as evaluation metrics. The analysis of included studies revealed no statistically significant differences in CR, PR, SD, or ORR between the group receiving combination therapy and the group receiving only chemotherapy (Supplementary Figure 2A–D). However, for DCR, the group treated with TCM and chemotherapy showed superior performance compared to the chemotherapy-only group (OR = 1.83; 95% CI: 1.07 − 3.12, *p* = 0.03, I^2^ = 0%, Figure 3A).

Median survival, along with the 1-year, 3-year, and 5-year survival rates, were utilized as metrics to assess the effectiveness of the herbal medicine combined with the chemotherapy group. As illustrated in Figure 3B, the median survival was notably higher in the TCM combination chemotherapy group compared to the chemotherapy alone group (OR = 2.91; 95% CI: 2.70–3.12, *p* < 0.00001, I^2^ = 90%). Furthermore, the collective findings indicated that the combination therapy group exhibited superior 1-year survival (OR = 3.18; 95% CI: 2.32–4.34, *p* < 0.00001, I^2^ = 58%, Figure 3C), 3-year survival (OR = 4.60; 95% CI: 2.99–7.06, *p* < 0.00001, I^2^ = 56%, Figure 3D), and 5-year survival (OR = 3.02; 95% CI: 2.14–4.26, *p* < 0.00001, I^2^ = 45%, Figure 3E) compared to the chemotherapy alone group.

### Quality of life and adverse events

**Figure 4.**
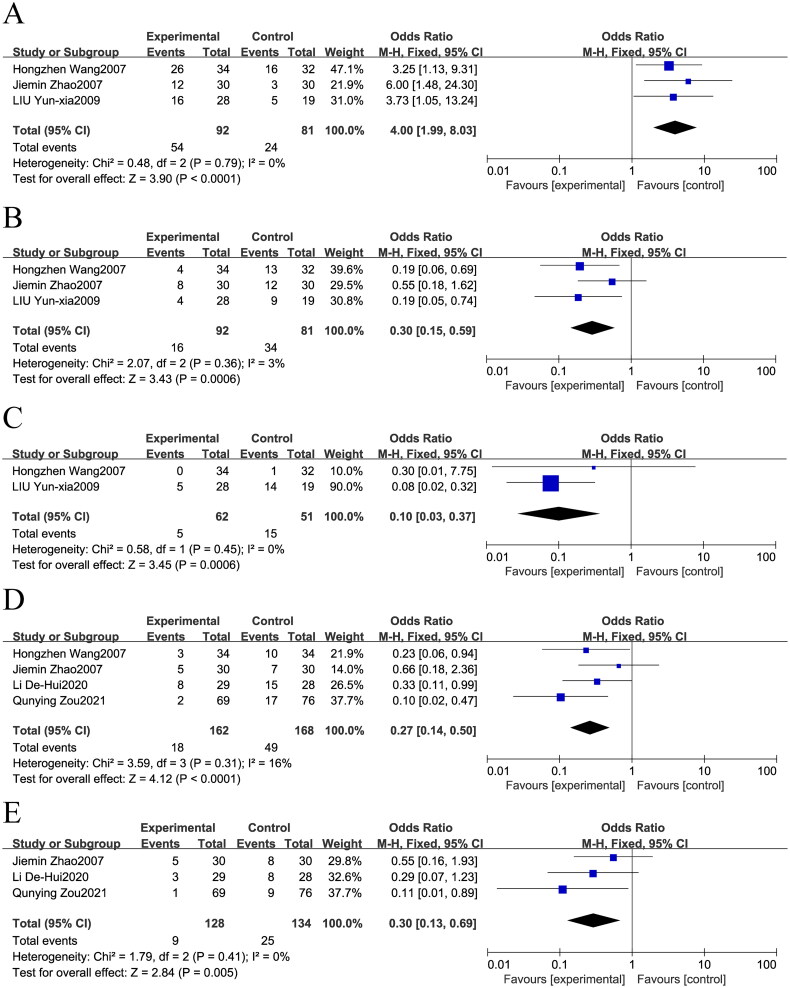
Forest plot of Chinese medicine combined with chemotherapy: (a): Qof improve; (B): Qof descend; (C): hemoglobin; (D): nausea and vomiting; (E): liver function injury.

Our research explored the effects of integrating TCM with chemotherapy compared to chemotherapy alone on the quality of patient survival. In this assessment, the group receiving combination therapy exhibited superior outcomes in improving life quality (OR = 4.00; 95% CI: 1.99–8.03, *p* < 0.0001, I^2^=0%, Figure 4A) and reducing quality of life deterioration (OR = 0.30; 95% CI: 0.15–0.59, *p* = 0.0006, I^2^ = 3%, Figure 4B). On the other hand, the group treated solely with chemotherapy showed no significant changes in quality-of-life stabilization (OR = 0.78; 95% CI: 0.38–1.60, *p* = 0.50, I^2^ = 0%, Supplementary Figure 2E).

In conclusion, data were compiled on adverse effects, which included reductions in erythrocytes, hemoglobin, and platelets, as well as incidences of nausea, vomiting, and hepatic impairment. Nonetheless, the combined results indicated that the occurrence of decreased erythrocytes and platelets in the combination therapy group did not significantly differ from that in the chemotherapy alone group (OR = 0.23; 95% CI: 0.05–1.08, *p* = 0.06, I^2^ = 69%, Supplementary Figure 2F; OR = 0.63; 95% CI: 0.16–2.48, *p* = 0.50, I^2^ = 0%, Supplementary Figure 2G). Conversely, significant disparities were observed in hemoglobin reduction (OR = 0.10; 95% CI: 0.03–0.37, *p* = 0.0006, I^2^ = 0%, Figure 4C), nausea and vomiting (OR = 0.27; 95% CI: 0.14–0.50, *p* < 0.0001, I^2^ = 16%, Figure 4D), and liver function impairment (OR = 0.30; 95% CI: 0.13–0.69, *p* = 0.005, I^2^ = 0%, Figure 4E) between the TCM with chemotherapy and chemotherapy-alone groups, with statistically significant differences favoring the combination therapy group. A power analysis was conducted to assess the sample size for the primary meta-analysis. The results showed that the power for most prognostic indicators of gastric cancer exceeded 0.6, indicating that our sample size was adequate. This further supports the robustness and reliability of our findings (Tables S5).

### Sensitivity analysis and publication bias test

The sensitivity analysis systematically removed each study included in the analysis one by one, and the aggregated analysis of the remaining studies was conducted to evaluate whether any single study disproportionately influenced the overall meta-analysis results. These sensitivity analysis findings are displayed in Supplementary Figure 3. The outcomes revealed that the majority of studies did not significantly skew the meta-analysis results, suggesting that the remaining studies’ outcomes were robust and dependable. Simultaneously, to mitigate the impact of Chinese medicine categories on our study, we categorized Chinese medicine treatments into two groups according to the guidelines [26]: tonifying therapies and digestive regulation therapies. Subgroup analysis was then conducted. Our findings, as depicted in Supplementary Figure 4 and Supplementary Figure 5, suggest that the classification of Chinese medicine did not significantly influence the treatment outcomes. Additionally, the funnel plot, presented in Supplementary Figure 6A-6H, displayed a symmetrical shape, indicating the absence of substantial publication bias in this study.

### Construction of TCM-GC network

**Figure 5.**
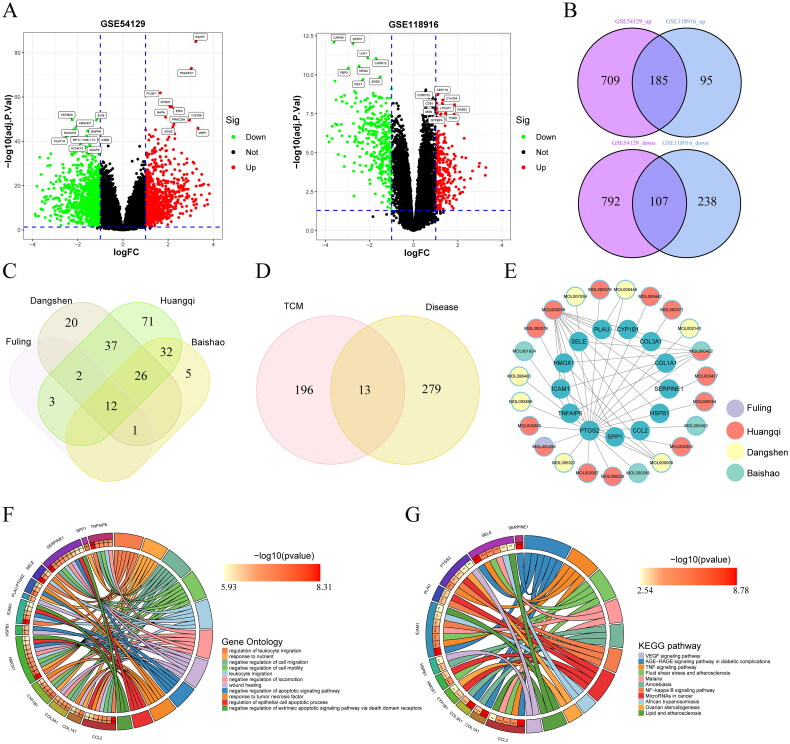
(A): Volcano plot of differential genes from GSE54129 and GSE118916 datasets; (B): Venn diagram of differential genes from GSE54129 and GSE118916 datasets; (C): intersection diagram of TCM drug target genes; (D): Venn diagram of differential genes between TCM treatments and GC; (E): network diagram of core genes and drug components; (F) GO analysis of core genes; (G) KEGG pathway analysis of core genes.

Initially, differential genes were obtained from two GC datasets (Figure 5A), and an intersection analysis was conducted on upregulated and downregulated differential genes (Figure 5B). These datasets collectively identified 292 GC genes, comprising 185 upregulated and 107 downregulated genes (Table S6). Analysis of the frequency of herb usage in TCM formulations documented in the literature was performed, revealing the most frequently used effective herbs such as Poria, Astragalus, Codonopsis, and Radix paeoniae alba (Table 2).

The formula database was queried, revealing that the four effective herbs contained a total of 44 compounds and 209 herb-target genes (Table S7). A Venn diagram was created to illustrate the overlap of drug-target genes for these herbs (Figure 5C), showing that multiple herbs may target the same genes. Functional enrichment analyses were then conducted separately for each of the four herbs, identifying the top 10 biological process (BP), cellular component (CC), and molecular function (MF) pathways, as well as the top 30 KEGG pathways (Supplementary Figure 7). Additional findings are presented in Table S8.

An intersection analysis of the four drug molecules and disease targets yielded 13 core TCM-GC genes (Figure 5D). A network map of the drug components and core genes was then constructed using Cytoscape 3.8.2 software (Figure 5E). GO analysis demonstrated associations of these genes with lymphatic invasion and cell migration (Figure 5F), and KEGG analysis linked them to the NF-κB and PI3K-AKT signaling pathways (Figure 5G).

### Cellular localization of key genes

**Figure 6.**
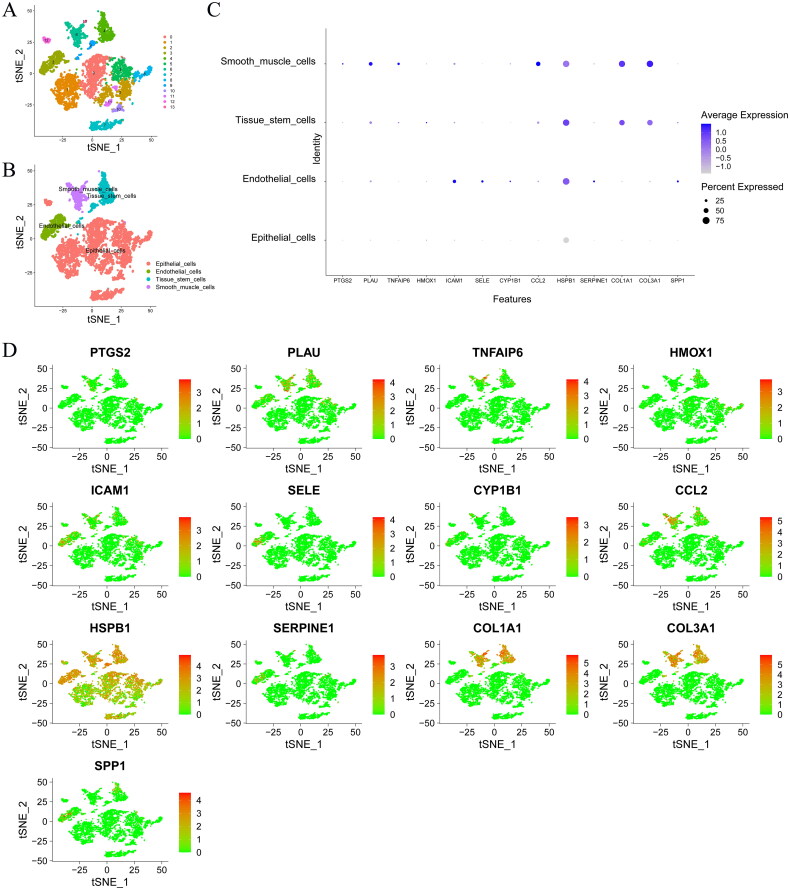
(A) Dimensionality reduction of cells in the GSE150290 GC dataset; (B): cell annotation in the GSE150290 dataset; (C): cellular distribution of core genes in gastric cancer cell populations; (D): single-cell gene expression of core genes.

Single-cell RNA sequencing facilitated the identification of cell types and the delineation of gene expression variations across various cell clusters. Employing unsupervised Seurat clustering analysis, 13 distinct cell clusters were recognized in GC (Figure 6A), which were primarily classified into four categories: epithelial cells, endothelial cells, tissue stem cells, and smooth muscle cells (Figure 6B). Analysis showed diverse expression patterns and proportions of core genes among these cell types (Figure 6C, D). Notably, HSPB1, COL1A1, and COL3A1 were predominantly expressed in smooth muscle cells and tissue stem cells, whereas ICAM1, SELE, and HSPB1 were highly expressed in endothelial cells.

### Cell communication investigation

**Figure 7.**
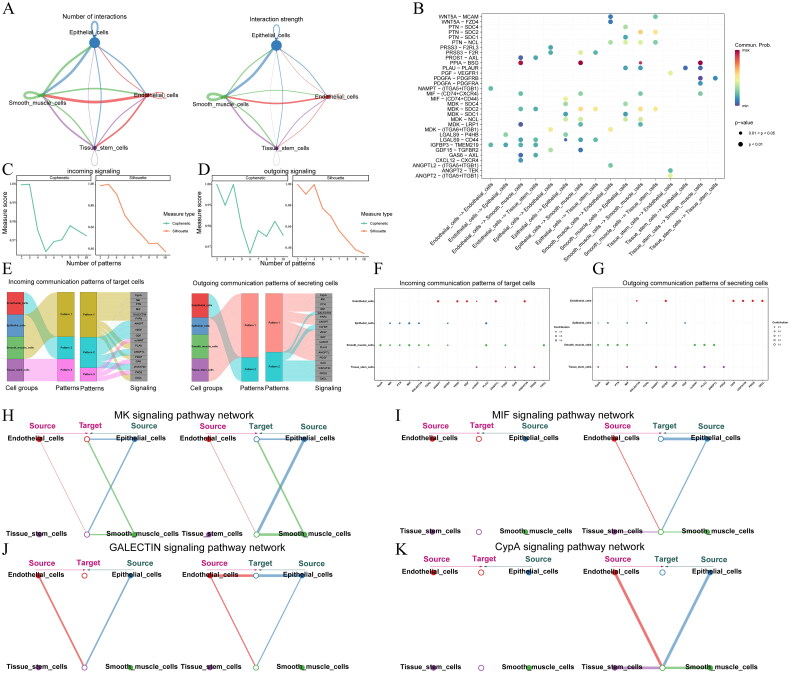
(A): The number and strength of interactions between different cell types are represented, where nodes indicate cell types and arrows indicate the direction of the relationship. The thickness of the lines correlates with the number or strength of interactions; (B): a bubble plot illustrating the expression of ligand-receptor pairs in cell-cell interactions. The x-axis represents interacting cells, and the y-axis represents specific pathways or ligands. The color of the dots indicates communication probability, and the size of the dots represents statistical significance, with larger dots indicating greater statistical difference. (C-D): incoming signaling and outgoing signaling. These plots use two different measure types: "cophenetic" (green) and "silhouette" (orange). The "cophenetic" score measures the accuracy of the clustering in representing the data’s hierarchical structure, with higher scores indicating better clustering quality. The "silhouette" score evaluates how similar an object is to its own cluster compared to other clusters, with scores ranging from -1 to 1; higher scores indicate better-defined clusters. (E): the incoming and outgoing patterns reveal how sending cells coordinate with other cells and synchronize with specific signaling pathways to drive communication. (F) a bubble plot of cell-pathway interactions in incoming communication patterns; (G) A bubble plot of cell-pathway interactions in outgoing communication patterns; (H) MK signaling pathway network; (I) MIF signaling pathway network; (J) GALECTIN signaling pathway network; (K) CypA signaling pathway network.

By analyzing scRNA-seq data, mechanisms of intercellular communication were explored. The cell − cell communication network diagram revealed extensive interactions between endothelial cells and epithelial cells with smooth muscle cells (Figure 7A, Supplementary Figure 8A). Bubble plots depicted relationships between different cells and specific pathways or ligands (Figure 7B), while heatmaps displayed the strength of outgoing and incoming signal pathways among different cells (Supplementary Figure 8B). Through non − negative matrix factorization (NMF), we identified communication patterns of cell incoming signaling and outgoing signaling, selecting the point of the first sharp drop as the pattern recognition criterion, namely incoming Pattern 3 (Figure 7C) and outgoing Pattern 2 (Figure 7D). Relationships between cells and various signal pathways were determined using river plots and heatmaps (Figure 7E, Supplementary Figure 8C, D), while bubble plots delineated specific incoming and outgoing signaling pathways with cells (Figure 7F, G), revealing important signal pathways such as cyclophilin A (CypA), Midkine (MK), PTN, Macrophage migration inhibitory factor (MIF), and GALECTIN. The association of specific signal pathways in different cells was depicted through hierarchical diagrams, providing insights into autocrine and paracrine actions. For instance, in the MK signaling pathway, epithelial cells served as the primary source of ligands and receptors, acting on themselves through autocrine and paracrine mechanisms (Figure 7H). In the MIF signaling pathway, epithelial cells acted on themselves through autocrine mechanisms, while in the GALECTIN signaling pathway, they acted on endothelial cells through paracrine mechanisms (Figure 7I, J). In the CypA signaling pathway, epithelial cells and endothelial cells acted on smooth muscle cells through autocrine and paracrine mechanisms (Figure 7K). Noteworthy ligand-receptor pairs in these pathways included PPIA-BSG, MDK-(ITGA6 + ITGAB1), MIF-(CD74 + CD44), and LGALS9-P4HB (Supplementary Figure 9).

## Discussion

TCM has been clinically used as a treatment method for GC [27]. A retrospective study by Hung et al. found that incorporating TCM into GC treatment significantly improves patient survival rates [28]. Similarly, Xu et al. demonstrated that TCM enhances the efficacy of GC treatments [29]. The pharmacological mechanisms of TCM are believed to involve the inhibition of epithelial-mesenchymal transition (EMT) [30–33] and cancer cell migration [34–36], the induction of cell cycle arrest [37,38], and apoptosis [39–41]. Moreover, chemotherapy-related side effects can significantly reduce the quality of life for GC patients. Platinum-based drugs, such as cisplatin, often cause severe gastrointestinal reactions and nephrotoxicity [42], while targeted drugs like apatinib can lead to severe mental exhaustion and drug-induced hypertension [43]. Studies have shown that combining TCM with chemotherapy can mitigate these side effects [44]. The multi-target characteristics of TCM may help avoid the over-regulation of a single target or signaling pathway, thereby reducing chemotherapy side effects. Previous retrospective studies have evaluated the therapeutic value of combining TCM with chemotherapy for GC [45,46]. However, these studies did not identify the optimal selection of TCM nor explore the potential molecular mechanisms involved. Therefore, our study aims to provide new evidence on the role of TCM combined with chemotherapy in treating GC, contributing to more personalized management of GC patients.

The meta-analysis results of this study indicated that, compared to chemotherapy alone, the combination of TCM and chemotherapy significantly improved the DCR and median survival of GC patients. These effects were consistently observed in subgroup analyses at 1, 3, and 5 years, aligning with the findings of several other studies. For example, Li et al. found that TCM combined with chemotherapy reduced recurrence and metastasis in advanced GC [47], while Shih et al. reported that both short-term and long-term use of TCM alongside chemotherapy prolonged survival rates [48]. Additionally, Liu et al. demonstrated that TCM combined with chemotherapy effectively improved the median survival time of advanced GC patients [8]. In terms of ORR, one study suggested that TCM combined with chemotherapy improved the ORR for GC [49], which contradicted our meta-analysis findings. This discrepancy might have been due to differences in retrieval strategies and follow-up periods, warranting further investigation. However, our study was consistent with others in showing that TCM reduced the side effects of chemotherapy and enhanced the quality of life for patients, as supported by the conclusions of Pan and Wilke et al. [50,51]. Although the results of the meta-analysis suggested that the classification of TCM was unrelated to the treatment outcomes for GC, the lack of meaningful results may have been due to the limited sample size in the studies after classification. Further clinical trials on the efficacy of TCM classifications in GC treatment would be needed in the future. Overall, these findings highlighted the clinical value of combining TCM with chemotherapy in the treatment of GC.

The meta-analysis results showed that Codonopsis, Poria, Radix paeoniae alba, and Astragalus are the high-frequency Chinese medicines used in chemotherapy for GC; these TCMs have been widely applied in the treatment of GC. A multicenter study by Shu et al. found that Yiqi Huayu Jiedu (containing Astragalus, Radix paeoniae alba, and poria) prolonged the disease-free survival (DFS) of GC patients and improved their quality of life [22]. Poria, a key component of the Sijunzi Decoction, had also been identified as effective in treating GC [52]. The anticancer mechanisms of these Chinese medicines can be summarized into two main points. First, they regulate molecular mechanisms related to GC to inhibit its development. For instance, Huo et al. found that Weikang Keli, a Chinese medicine containing Codonopsis, induces autophagy-mediated cell death in GC cells [53]. He et al. reported that Codonopsis regulates cellular energy metabolism, thereby inhibiting GC proliferation [54]. Cheng et al. found that extracts of Codonopsis downregulate the ASCT2, inhibiting GC cell proliferation [55]. Wang et al. discovered that Poria combined with chemotherapy drugs effectively inhibits the EMT of GC [56]. Xie et al. found that Poria extracts regulate the MAPK/PI3K pathway to inhibit the proliferation of GC [57]. Additionally, *in vitro* experiments revealed that Poria acid can inhibit the invasion and metastasis of GC [58]. Na et al. found that Astragalus extract alleviates damage to mesothelial cells caused by GC through apoptosis [59]. Yu et al. discovered that Astragalus extract kills GC cells by inducing mitochondrial apoptosis [60]. Second, TCM enhances the efficacy of chemotherapy drugs. Chen et al. found that Astragalus, when combined with platinum-based chemotherapy drugs, increases their therapeutic effect in patients [61]. Tan et al. showed that Astragalus and Codonopsis enhance the response of oxaliplatin in advanced GC [62]. To explore the potential mechanisms by which these TCMs affect chemotherapy outcomes, KEGG analysis was performed on individual TCMs and intersection genes with TCM-GC. The results suggested that the NF-κB and PI3K-AKT pathways were involved, consistent with other studies. For instance, Astragalus polysaccharide extracts were shown to inhibit the AKT pathway in GC and enhance the antitumor effect of Apatinib [63]. Paeoniflorin, an extract from Paeoniae alba [64], was found by Wu et al. to inhibit the NF-κB pathway, thereby enhancing the efficacy of 5-fluorouracil in killing GC cells [65].

A total of 13 TCM-GC targets were identified in this study. Single-cell sequencing analysis suggested that some targets, such as ICAM1, SELE, HSPB1, and SPP1, were associated with gastric endothelial cells and might be related to GC. This hypothesis is supported by several studies. Single-cell analyses revealed that the expression of several key genes differed across various gastric cancer (GC) subtypes, which may have been closely associated with the progression of the disease. Several studies provided a theoretical foundation for this observation. Chen et al. found that the downregulation of HSPB1 enhanced autophagy in smooth muscle cells [66], and increased autophagic activity was linked to the progression of gastric cancer and drug resistance [67,68]. Zheng et al. reported that the downregulation of COL1A1 was associated with smooth muscle cell viability and inhibited cell proliferation [69]. Yoo et al. demonstrated that soluble ICAM1 activated endothelial cells in gastric cancer and was associated with disease progression [70]. Li et al. highlighted that ICAM1 expression in endothelial cells was closely related to inflammation and promoted gastric cancer development [71]. Chen et al. found that ICAM1 might be involved in the development of GC [72], with its expression linked to metastasis [73–76]. Jung et al. reported that targeting ICAM1 could enhance T cell function in killing GC cells [77]. Benekli et al. found that serum SELE levels are associated with CEA and poor prognosis in GC [78]. Jiang et al. experimentally demonstrated that the Chinese herb Andrographis paniculata inhibits the adhesion of endothelial cells to GC cells by decreasing SELE expression [79]. Peng et al. discovered that the FYN/TOPK/HSPB1 axis is closely related to the proliferation and metastasis of GC [80]. Song et al. found that the upregulation of SPP1 activates the PI3K/AKT pathway, leading to chemoresistance in GC [81]. Additionally, Du et al. demonstrated that SPP1 is involved in the immunoregulation of GC [82]. The aforementioned mechanisms highlight the value of these genes as targets for GC. However, further experimental validation is needed to elucidate the regulatory mechanisms of these targets in GC.

The results of cell communication analysis suggested that certain pathways were involved in endocrine regulation between different GC cells. Rha et al. found that the MK signaling pathway exhibited both autocrine and paracrine activities in GC, which was consistent with our findings and correlated with tumor size [83]. A survey of serum and urine MK levels in Chinese individuals conducted by Huang’s team showed that MK expression levels in GC tissues correlate with both stage and metastasis [84]. Additionally, a retrospective study by Zhao et al. found that high MK expression is associated with poor prognosis in GC [85]. Subsequent mechanistic studies revealed that upregulated MK can regulate mitochondrial polarization to promote cell growth [86], suppress NK cell cytotoxicity [87], and enhance cisplatin resistance in GC through the Notch signaling pathway [88]. The MIF signaling pathway was also involved in the regulation of GC, as supported by several studies. He et al. found that serum MIF levels were elevated in GC compared to normal mucosa [89]. Kong et al. discovered that the MIF/P53 regulatory axis promoted the progression of GC [90]. The results of the present study suggested a correlation with the CD74 receptor. The potential of CD74 and MIF as therapeutic targets for GC was subsequently confirmed experimentally by Zheng et al. [91]. The regulatory mechanism of the GALECTIN signaling pathway in GC has not yet been fully determined. However, studies found associations between various galectins and different aspects of GC metastasis and proliferation. Specifically, galectin-1, -3, and -4 were associated with peritoneal metastasis [92–95], galectin-2 with lymphatic metastasis [96], and galectin-9 with the proliferation of GC [97]. These findings suggested the potential value of the GALECTIN signaling pathway as a therapeutic target, warranting further experimental investigation in the future. Finally, regarding the CypA signaling pathway, Feng et al. found that elevated CypA levels were associated with the proliferation of GC [98]. *In vitro* experiments showed that inhibiting CypA reduced the proliferation of GC cells through MAPK-related pathways [99]. Subsequent studies by Han et al. confirmed the value of CypA as a therapeutic target for GC [100].

This study has several limitations. First, the meta-analysis included studies that employed diverse treatment strategies for GC, with variations in sample characteristics potentially contributing to heterogeneity. To mitigate this issue, future research should prioritize collaboration with other hospitals to increase sample sizes and implement rigorous inclusion criteria, both of which would help reduce heterogeneity. Moreover, while subgroup analyses were performed based on different types of TCM, future studies with larger cohorts should extend these analyses to include additional clinical characteristics. The primary objective of this study was to evaluate the prognostic value of combining TCM with chemotherapy in GC. Developing precise and individualized TCM strategies will be a key focus of future research. Lastly, although network pharmacology and cell-cell communication analyses identified several critical targets and pathways, these findings require further validation through *in vitro* experiments to confirm their underlying mechanisms.

## Conclusion

Despite employing various approaches, including meta-analysis, network pharmacology, single-cell sequencing, and cell-cell communication analysis, this study has several limitations. First, heterogeneity among the included studies may have influenced the accuracy of the results. Second, while we predicted the potential mechanisms of TCM combined with chemotherapy for treating gastric cancer, these findings require further experimental validation to confirm their clinical significance. Moreover, although this study identified key targets and signaling pathways associated with the combination of TCM and chemotherapy, further investigation is needed to elucidate the underlying mechanisms and develop clearer therapeutic strategies for clinical application. Lastly, while this study provides preliminary evidence supporting the potential of TCM combined with chemotherapy in gastric cancer treatment, its translation into clinical practice remains challenging. Future research should focus on large-scale clinical trials and robust sample data to validate the efficacy and safety of this therapeutic approach. With advancements in precision medicine and individualized treatment, integrating TCM into gastric cancer chemotherapy could offer improved outcomes for patients.

## Supplementary Material

PRISMA_2020_checklist.docx

## Data Availability

The original contributions presented in the study are included in the article/Supplementary Material. Further inquiries can be directed to the corresponding author.
